# Gender differences in learning and study strategies impact medical students’ preclinical and USMLE step 1 examination performance

**DOI:** 10.1186/s12909-024-05494-z

**Published:** 2024-05-07

**Authors:** Sparsha Saxena, William S Wright, Mohammed K. Khalil

**Affiliations:** 1 University of South Carolina School of Medicine, 29605 Greenville, SC USA; 2 Department of Biomedical Sciences, University of South Carolina School of Medicine, 29605 Greenville, SC USA

**Keywords:** Learning strategies, LASSI, USMLE Step 1, Preclinical performance, Gender differences, Anxiety

## Abstract

**Background:**

Evaluation of students’ learning strategies can enhance academic support. Few studies have investigated differences in learning strategies between male and female students as well as their impact on United States Medical Licensing Examination® (USMLE) Step 1 and preclinical performance.

**Methods:**

The Learning and Study Strategies Inventory (LASSI) was administered to the classes of 2019–2024 (female (*n* = 350) and male (*n* = 262)). Students’ performance on preclinical first-year (M1) courses, preclinical second-year (M2) courses, and USMLE Step 1 was recorded. An independent t-test evaluated differences between females and males on each LASSI scale. A Pearson product moment correlation determined which LASSI scales correlated with preclinical performance and USMLE Step 1 examinations.

**Results:**

Of the 10 LASSI scales, Anxiety, Attention, Information Processing, Selecting Main Idea, Test Strategies and Using Academic Resources showed significant differences between genders. Females reported higher levels of Anxiety (*p* < 0.001), which significantly influenced their performance. While males and females scored similarly in Concentration, Motivation, and Time Management, these scales were significant predictors of performance variation in females. Test Strategies was the largest contributor to performance variation for all students, regardless of gender.

**Conclusion:**

Gender differences in learning influence performance on STEP1. Consideration of this study’s results will allow for targeted interventions for academic success.

**Supplementary Information:**

The online version contains supplementary material available at 10.1186/s12909-024-05494-z.

## Background

Various approaches to learning and studying contribute to success in medical school. Prior studies have found associations between certain learning and study strategies and success within the preclinical environment as well as on specific standardized examinations, such as the United States Medical Licensing Examination® (USMLE) Step 1. However, research thus far [1] has only explored patterns within the entire student body. There is a current gap in our understanding of whether certain student characteristics, such as gender, lead to different learning and study strategies and whether those differences influence performance.

Learning and study strategies are the approaches students use to acquire new course content [2]. There are numerous strategies that can be studied, such as, preferences for didactic versus problem-based lectures [3] and/or applying knowledge through experiences versus abstraction and conceptualization [4]. Additionally, student characteristics including demographics such as gender or age, academic background, social and emotional components, and cognitive characteristics also influence learning and study strategy utilization [5].

Surveys and questionnaires are instruments that can identify which strategies students use. In addition to finding patterns toward content acquisition or learner characteristics such as motivation or self-esteem [6], data from questionnaires can provide insight on which approaches to learning, as well as, what emotional characteristics have the greatest impact in overall performance [6, 7].

The Learning and Study Strategies Inventory (LASSI) is a questionnaire that evaluates students in the domains of skill, will and self-regulation as they apply to learning [8]. The skill domain measures techniques of content acquisition, and it is described through scores on the scales of Information Processing, Selecting Main Idea, and Test Strategies. For example, it looks at whether a student can understand novel information, identify the salient points, and ultimately apply that knowledge in a testing situation. The will domain is described through scores on the scales of Attitude, Motivation, and Anxiety [8]. It reflects how one’s emotional characteristics, whether positive or negative, impacts how they learn. Finally, the self-regulation domain is described through scores on the scales of Concentration, Time Management, Self-Testing, and Using Academic Resources [8]. The self-regulation domain measures the degree to which students control their learning environment through focus, appropriate allocation of time, and effective use of resources such as teachers or peers.

LASSI results have shed light on how scores on certain scales impact medical student performance. In assessing performance during the first semester of medical school, investigators found Time Management and Self-Testing, both components of the self-regulation domain, were stronger predictors of academic performance than aptitude, as measured by the Medical College Admissions Test (MCAT) and undergraduate Grade Point Average (GPA) [9]. Similarly, LASSI scales of Anxiety, Selecting Main Idea, and Test Strategies significantly correlated with performance on the USMLE Step 1 and overall preclinical grades [1]. Furthermore, when comparing academically high performing students to low-performing students, Anxiety, Motivation, and Test Strategies scores were significantly different between the top and bottom quartiles [10]. There are numerous other studies that have shown LASSI scales can help explain performance differences on examinations outside of the medical school environment [11–13]. Consequently, scores on LASSI scales provide objective data on learning and study strategies that can help students and faculty target specific areas for improving overall student performance.

The available analysis on LASSI results and medical school performance aggregates the student body as a whole. However, medical school is composed of increasingly diverse student populations and there may be unidentified trends in learning and study approaches when comparing students sharing similar characteristics, such as gender or those who are first-generation to those who are non-traditional students. Understanding the differences that occur will help guide where counseling and supportive resources are needed. This study investigates LASSI scores between male and female medical students across LASSI scales. It also explores if differences impact performance during the preclinical years as well as on the USMLE Step 1.

## Methods

### Design and participants

The study was a retrospective repeated cross-sectional study, which provided longitudinal data for analysis. Medical students completed the LASSI mid-way through their first year of medical school. Students were required by the Academic Success Program to complete LASSI for both diagnostic and prescriptive measures. The sample consisted of 618 medical students (350 females, 262 males, 6 did not identify as either male or female) across six classes (classes of 2019–2024) in their preclinical years at the University of South Carolina School of Medicine Greenville (USCSOMG). Participants were 55.6% females and 44.4% males. Their ages range from 20 to 45 years with an average age of 23 years. Students’ pre-matriculation characteristics included an average MCAT of 68th percentile, and an average undergraduate GPA of 3.68 on a 4-point scale. Ethics approval and informed consent is exempted by the University of South Carolina Institutional Review Board (IRB) (Ref. #: Pro00111001).

### Instrument

The LASSI 3rd edition is a 60-item inventory that contains 6 items for each of the 10 LASSI scales. Description of the LASSI 10-scale by Weinstein et al. (2016) is summarized in Table 1. Participants answer each item on a 5-point Likert scale wherein 1 = not at all typical of me, 2 = not very typical of me, 3 = somewhat typical of me, 4 = fairly typical of me, and 5 = very much typical of me. Participants receive percentile scores for each LASSI scale. Higher percentile scores in a LASSI scale correspond with strength in that domain, whereas lower scores in a scale correspond with need for improvement in that area. For example, a high score in Anxiety can be interpreted as someone for whom anxiety levels or stress does not hinder learning. But a low score in Anxiety signifies that the student struggles with anxiety in the learning environment. The reliability of LASSI 10 scales is measured by Cronbach Alpha of 0.76–0.87, and demonstrates good validity [8].

**Table 1:** The Scale and its description for the LASSI

Scale	Description
ANX	Anxiety and worry about school performance
ATT	Attitude and interest
CON	Concentration and attention to academic tasks
INP	Information processing, acquiring knowledge, and reasoning
MOT	Motivation, diligence, self-discipline, and willingness to work hard
SMI	Selecting main ideas and recognizing important information
SFT	Self-testing, reviewing, and preparing for classes
TST	Test strategies and preparing for tests
TMT	Use of time management principles for academic tasks
UAR	Using academic resources available to students

Note: ANX, Anxiety; ATT, Attitude; CON, Concentration; INP, Information Processing; MOT, Motivation; SMI, Selecting Main Ideas; SFT, Self-Testing; TST, Test Strategies; TMT, Time Management; UAR, Using Academic Resources

### Educational context

The pre-clerkship curriculum at the USCSOMG integrates basic and clinical sciences. In the first year of the medical school (M1), the foundational basic sciences are taught in the following modules: Foundations of Medicine, Structure and Function of the Human Body Part 1 & 2, Neuroscience, and Defenses & Responses. Beside these modules, the Integrated Practice of Medicine (IPM) module is delivered throughout M1 year to promote clinical reasoning skills. In the second year (M2), the curriculum teaches the mechanism and management of diseases in the organ-based modules (Biomedical Principles of Disease Therapy, Hematology/Oncology, Mind, Brain & Behavior, Cardiovascular/Pulmonary/Renal, GI/Hepatic, Endocrine & Reproductive, and Musculoskeletal/Dermatology/Rheumatology). Similar to M1 year, IPM module is delivered across the entire M2 year.

### Data collection

The LASSI instrument was administered to the 618 medical students following their first semester of medical school and their scores at this time were recorded. Weighted average of students’ performance during the first year of medical school (M1), preclinical weighted average, and USMLE Step 1 examination scores were collected for all participants. However, USMLE Step 1 scores for the class of 2024 were not analyzed as scores were given as Pass/Fail. For gender differences, LASSI scores were divided into male and female scores.

### Statistical analysis

International Business Machines® (IBM) Statistical Package for the Social Sciences® (SPSS) was used to perform data analysis. LASSI scale scores between male and female students were compared using an independent t-test. Pearson Product-Moment correlation was performed to measure the strength of association between LASSI scores and M1 weighted average, preclinical weighted average and USMLE Step 1 performance. The r-squared value is presented and it provides the percent of the dependent variable that is explained by the independent variable. For example, the LASSI scale of Anxiety explains 8.64% of the M1 preclinical performance (Table 2).

**Table 2:** Strength of association between the ten LASSI scales and performance on M1 weighted average, preclinical weighted average, and USMLE Step 1 for all students

Scale	M1 Average		Preclinical Average		USMLE Step 1	
	(*N* = 612)		(*N* = 584)		(*N* = 484)	
	*r*^2^ value	*p* value	*r*^2^ value	*p* value	*r*^2^value	*p* value
ANX	8.64%	< 0.001***	4.20%	< 0.001***	7.18%	< 0.001***
ATT	2.40%	< 0.001***	2.04%	0.001**	0.16%	0.376
CON	9.86%	< 0.001***	9.18%	< 0.001***	4.62%	< 0.001***
INP	4.33%	< 0.001***	2.59%	< 0.001***	3.28%	< 0.001***
MOT	18.23%	< 0.001***	17.22%	< 0.001***	6.50%	< 0.001***
SMI	6.97%	< 0.001***	5.15%	< 0.001***	6.40%	< 0.001***
SFT	11.90%	< 0.001***	10.43%	< 0.001***	7.73%	< 0.001***
TST	18.92%	< 0.001***	13.32%	< 0.001***	13.47%	< 0.001***
TMT	11.63%	< 0.001***	11.49%	< 0.001***	4.16%	< 0.001***
UAR	0.49%	0.083	0.44%	0.11	0.28%	0.245

Note: ANX, Anxiety; ATT, Attitude; CON, Concentration; INP, Information Processing; MOT, Motivation; SMI, Selecting Main Ideas; SFT, Self-Testing; TST, Test Strategies; TMT, Time Management; UAR, Using Academic Resources

*p < 0.05, **p < 0.01, ***p < 0.001 (2-tailed)

## Results

The results indicate that among the 10 scales for the LASSI, the five highest scores across all classes were observed in Anxiety, Attitude, Motivation, Test Strategies, and Time Management (Table 3). Using Academic Resources scale had the lowest score overall (Table 3). Anxiety, Attitude, Information Processing, Selecting Main Ideas, Self-Testing, Test Strategies, and Using Academic Resources had significant differences in mean scores between males and females (Table 3). Concentration, Motivation, and Time Management showed no significant score differences between genders (Table 3). Females scored significantly higher on Attitude and Using Academic Resources (Table 3). Males scored significantly higher on Anxiety, Information Processing, Selecting Main ideas, Self-Testing, and Test Strategies (Table 3).

**Table 3:** Gender difference in the percentage of mean scores of the ten LASSI scales

Scale	All Students (*N* = 618)	Males (*N* = 262)	Females (*N* = 350)	t	*P*
	Mean (SD)	Mean (SD)	Mean (SD)		
ANX	65.01 (26.92)	73.45 (22.11)	59.18 (28.30)	6.765	< 0.001***
ATT	64.59 (24.87)	59.31 (26.93)	68.73 (22.48)	-4.711	< 0.001***
CON	54.47 (27.96)	54.79 (27.53)	54.46 (28.20)	0.148	0.883
INP	61.00 (26.92)	64.94 (25.81)	58.42 (27.23)	2.996	0.003**
MOT	62.55 (26.37)	61.86 (26.14)	63.29 (26.52)	− 0.664	0.507
SMI	56.44 (27.96)	61.96 (25.91)	52.69 (28.78)	4.114	< 0.001***
SFT	55.74 (27.63)	58.92 (27.01)	53.64 (28.02)	2.343	0.019*
TST	63.96 (25.59)	67.61 (24.13)	61.53 (26.27)	2.932	0.003**
TMT	64.27 (29.19)	65.31 (28.26)	63.72 (30.02)	0.665	0.506
UAR	53.64 (28.75)	45.50 (27.59)	59.69 (28.04)	-6.237	< 0.001***

Note: ANX, Anxiety; ATT, Attitude; CON, Concentration; INP, Information Processing; MOT, Motivation; SMI, Selecting Main Ideas; SFT, Self-Testing; TST, Test Strategies; TMT, Time Management; UAR, Using Academic Resources. SD, Standard Deviation

*p < 0.05, **p < 0.01, ***p < 0.001 (2-tailed)

Note: USMLE Step 1 Scores not included from Class of 2024 because results were provided as P/F.

For all classes combined, apart from Attitude and Using Academic Resources, LASSI scales were significantly associated with all academic performance measures: M1 weighted average, preclinical weighted average, and USMLE Step 1 examination scores (Table 2). All ten scales, except for Using Academic Resources, were found to have significant correlation with M1 performance (Table 2). For the overall preclinical performance, the same outcome, as seen with M1 performance was observed (Table 2). USMLE Step 1 performance showed significant correlation with all ten scales except for Attitude and Using Academic Resources (Table 2).

Furthermore, for all students, Test Strategies contributed a significant amount of variation in performance across M1, preclinical grades, and USMLE Step 1 (Tables 2, 4, 5 and 6). Additionally, Motivation, Self-Testing, and Time Management explain more than 10% of the variations in M1 weighted average and preclinical weighted average (Table 2).

**Table 4:** M1 weighted average score variation explained by the scores of the ten LASSI scales

Scale	All Students		Males		Females	
	(*N* = 612)		(*N* = 261)		(*N* = 347)	
	*r*^2^ value	*p* value	*r*^2^ value	*p* value	*r*^2^ value	*p* value
ANX	8.64%	< 0.001***	4.28%	< 0.001***	9.73%	< 0.001***
ATT	2.40%	< 0.001***	1.30%	0.065	5.11%	< 0.001***
CON	9.86%	< 0.001***	3.50%	0.002**	14.67%	< 0.001***
INP	4.33%	< 0.001***	1.14%	0.086	6.05%	< 0.001***
MOT	18.23%	< 0.001***	14.67%	< 0.001***	20.79%	< 0.001***
SMI	6.97%	< 0.001***	4.88%	< 0.001***	7.56%	< 0.001***
SFT	11.90%	< 0.001***	8.53%	< 0.001***	13.47%	< 0.001***
TST	18.92%	< 0.001***	10.89%	< 0.001***	23.14%	< 0.001***
TMT	11.63%	< 0.001***	5.66%	< 0.001***	16.48%	< 0.001***
UAR	0.49%	0.083	0.21%	0.462	1.54%	0.021*

Note: ANX, Anxiety; ATT, Attitude; CON, Concentration; INP, Information Processing; MOT, Motivation; SMI, Selecting Main Ideas; SFT, Self-Testing; TST, Test Strategies; TMT, Time Management; UAR, Using Academic Resources

*p < 0.05, **p < 0.01, ***p < 0.001 (2-tailed)

**Table 5:** Preclinical weighted average score variation explained by the scores of the ten LASSI scales

Scale	All Students		Males		Females	
	(*N* = 584)		(*N* = 254)		(*N* = 328)	
	*r*^2^ value	*p* value	*r*^2^ value	*p* value	*r*^2^ value	*p* value
ANX	4.20%	< 0.001***	1.32%	0.068	6.55%	< 0.001***
ATT	2.04%	0.001**	1.04%	0.105	3.61%	0.001**
CON	9.18%	< 0.001***	3.57%	0.002**	14.52%	< 0.001***
INP	2.59%	< 0.001***	0.92%	0.127	3.80%	< 0.001***
MOT	17.22%	< 0.001***	11.02%	< 0.001***	22.37%	< 0.001***
SMI	5.15%	< 0.001***	3.46%	0.003**	6.71%	< 0.001***
SFT	10.43%	< 0.001***	8.47%	< 0.001***	11.76%	< 0.001***
TST	13.32%	< 0.001***	9.00%	< 0.001***	16.56%	< 0.001***
TMT	11.49%	< 0.001***	5.38%	< 0.001***	16.97%	< 0.001***
UAR	0.44%	0.11	0.35%	0.348	0.92%	0.081

Note: ANX, Anxiety; ATT, Attitude; CON, Concentration; INP, Information Processing; MOT, Motivation; SMI, Selecting Main Ideas; SFT, Self-Testing; TST, Test Strategies; TMT, Time Management; UAR, Using Academic Resources

*p < 0.05, **p < 0.01, ***p < 0.001 (2-tailed)

**Table 6:** USMLE Step 1 score variations explained by the scores of the ten LASSI scales

Scale	All Students		Males		Females	
	(*N* = 484)		(*N* = 209)		(*N* = 274)	
	*r*^2^ value	*p* value	*r*^2^ value	*p* value	*r*^2^ value	*p* value
ANX	7.18%	< 0.001***	2.59%	0.020*	9.18%	< 0.001***
ATT	0.16%	0.376	0.27%	0.453	2.59%	0.008**
CON	4.62%	< 0.001***	0.66%	0.244	9.61%	< 0.001***
INP	3.28%	< 0.001***	1.54%	0.072	4.20%	0.001**
MOT	6.50%	< 0.001***	2.69%	0.018*	10.82%	< 0.001***
SMI	6.40%	< 0.001***	4.67%	0.002**	6.50%	< 0.001***
SFT	7.73%	< 0.001***	6.35%	< 0.001***	8.01%	< 0.001***
TST	13.47%	< 0.001***	7.62%	< 0.001***	17.14%	< 0.001***
TMT	4.16%	< 0.001***	0.55%	0.285	8.24%	< 0.001***
UAR	0.28%	0.245	0.98%	0.155	0.05%	0.711

Note: ANX, Anxiety; ATT, Attitude; CON, Concentration; INP, Information Processing; MOT, Motivation; SMI, Selecting Main Ideas; SFT, Self-Testing; TST, Test Strategies; TMT, Time Management; UAR, Using Academic Resources

*p < 0.05, **p < 0.01, ***p < 0.001 (2-tailed)

Note: USMLE Step 1 scores for class of 2024 not included in analysis because scores were provided as Pass/Fail.

When assessing the impact of LASSI scores by gender, variations in academic performance for female students, measured by the M1 weighted average (13.47–23.14%), preclinical weighted average (11.76–22.37%), were explained more by Concentration, Motivation, Self-Testing, Test Strategies, and Time Management as compared to male students (Tables 4 and 5). Score variations explained by Anxiety was also higher in females (9.18%) than males (2.59%) for USMLE Step 1 examinations (Table 6). Additionally, Motivation and Test Strategies had similar differences in explaining performance variation for male and female students on the USMLE Step 1 examination (Table 6).

In summary, the study revealed that the Anxiety scale had significantly different scores between males and females, with females having lower scores. It contributed to high variation in all performance measures for females only. Concentration, Motivation, and Time Management did not have significant differences between males and females, but these scales explain performance variation more for females. Finally, Testing Strategies was the most significant scale for both males and females in all performance measures.

## Discussion

The results confirm the initial hypothesis that gender-based differences in learning and study strategies exist in medical students and these differences impact performance in both the preclinical curriculum and on the USMLE Step 1 examination.

When LASSI 10-scale scores were evaluated based on gender, different findings emerged. Even though Anxiety was the highest score on average for all students, evaluation of male and female scores separately showed that females scored significantly lower than males, which indicates anxiety is hindering academic performance, to a larger degree, in females. The analysis revealed Anxiety contributed to variation in all performance measures for females.

Anxiety is widespread in the medical school environment. One in three medical students report increased levels of anxiety [14]. This may be due to the psychologically and academically rigorous environment of medicine, where time to relax and pursue hobbies freely is limited [14]. Prior studies seeking to understand anxiety and depression’s role in academic performance show an inverse relationship between anxiety and depression levels with levels of academic performance [15, 16]. That is, increased levels of anxiety are detrimental to academic success.

In line with our results, other studies within medical schools have shown anxiety to be more prevalent in females [17–19]. These findings support the need for medical schools to play a larger role in anxiety management for students, with special attention to females. As our results show, anxiety negatively impacts performance for females and properly addressing sources of anxiety and providing techniques to manage stress could improve outcome for this student population.

Early identification of students experiencing increased levels of anxiety in the academic environment can be a starting point to providing support. Results from LASSI may help identify students struggling with anxiety or school administrators can take an extra moment to ask about a student’s mental health. Medical schools can promote and encourage anxiety-reduction strategies for their students, such as encouraging regular physical [20], integration of yoga and meditation into the learning environment in between or following lectures [21], and simulated examinations or targeted study material to curb fear of test taking or poor performance [22]. Routine follow-up with students with attention to their mental health is vital in measuring whether interventions are successful [23]. In addition to improving academic outcomes, addressing anxiety can improve quality of life, well-being, and abate burn out for medical students [24].

Furthermore, even though males and females did not differ in the scales of Concentration, Motivation, and Time Management, these scales were important for females as they contributed to performance variations for all performance measures. For example, for STEP 1 examination, Concentration, Motivation, and Time Management contributed to 9.61%, 10.82%, and 8.24% of score variation, respectively. There is limited available data that explains why these factors impact females more than males. It may suggest that females differ in academic and emotional characteristics which influence performance. Correlational studies have found that women express higher levels of motivation toward academic achievement in comparison to men [25, 26]. While it is unclear why females are more motivated, it plays a role in their performance and may be leveraged to help them succeed on their examinations. Research in a broader academic context has found that motivational and attention factors strengthen the actions taken to achieve said goal [27]. More research on how these scales impact performance for women is welcomed.

Finally, this study showed that test strategies were critical for performance for males and females. It contributed most to performance variation for all students, regardless of gender, across all performance measures. The Test Strategies scale on the LASSI assesses how students prepare for examinations depending on the course and if they adequately review areas of weakness. An avenue that can assist in enhancing preparation for students can be through increased practice questions and simulated examination environments [28]. Classes that offer multiple opportunities for practice with interleaved lecture questions, post-lecture questions, and links to helpful reading resources not only improve examination performance but also student satisfaction [29]. Additionally, studies have found that increased practice problem completion, greater than at least 2000 in one study and 3500 in another, had significantly resulted in higher Step 1 scores [30, 31]. Another means that can aid in targeted preparation for examinations is through having high-performing students who have taken the classes and examinations tutor their peers, so they are able to provide specific advice for testing strategies. Preliminary studies of this approach have yielded higher performance on USMLE Step 1, Step 2, and medical school examinations for students who received effective tutoring [32].

The study is limited by the analyses of one institution’s data. Analyzing data from multiple institutions would strengthen the association that student characteristics influence learning and study strategies. Overall, this study shows that the approach may lead to missing key patterns that can assist in individualizing student support. Analyses of additional student characteristics such as race, first-generation status, non-traditional, or minority status could also reveal different approaches to learning.

In conclusion, the results revealed that among females, Anxiety, Concentration, Motivation, and Time Management influenced preclinical and STEP 1 performance. This was not seen for male students. For all students, Test Strategies had the largest influence in all performance measures. Recognition of different student characteristics allowed for more meaningful analysis of LASSI data. In turn, these findings can help create targeted interventions for improving academic support and pave the way for future research considering how student characteristics impact learning.

### Electronic supplementary material

Below is the link to the electronic supplementary material.

Supplementary Material 1

## Data Availability

The datasets used and/or analyzed during the current study are available from the corresponding author on reasonable request.
